# A longitudinal analysis of conspiracy beliefs and Covid-19 health responses

**DOI:** 10.1017/S0033291722002938

**Published:** 2023-09

**Authors:** Jan-Willem van Prooijen, David M. Amodio, Arnout Boot, Anita Eerland, Tom Etienne, André P. M. Krouwel, Michal Onderco, Peter Verkoeijen, Rolf A. Zwaan

**Affiliations:** 1 Department of Experimental and Applied Psychology, Vrije Universiteit Amsterdam, Amsterdam, the Netherlands; 2 The Netherlands Institute for the Study of Crime and Law Enforcement (NSCR), Amsterdam, the Netherlands; 3 Department of Criminal Law and Criminology, Maastricht University, Maastricht, the Netherlands; 4 Department of Psychology, New York University, New York, NY, USA; 5 Department of Social Psychology, University of Amsterdam, Amsterdam, the Netherlands; 6 Department of Psychology, Education, and Child Studies, Erasmus University Rotterdam, Rotterdam, the Netherlands; 7 Department of Communication Science, Radboud University Nijmegen, Nijmegen, the Netherlands; 8 Kieskompas, Amsterdam, the Netherlands; 9 Department of Political Science & Annenberg School for Communication, University of Pennsylvania, Philadelphia, PA, USA; 10 Departments of Political Science and Communication Science, Vrije Universiteit Amsterdam, Amsterdam, the Netherlands; 11 Department of Public Administration and Sociology, Erasmus University Rotterdam, Rotterdam, the Netherlands; 12 Peace Research Center Prague, Faculty of Social Sciences, Charles University, Prague, Czechia; 13 Brain and Learning Research Group, Learning and Innovation Center, Avans University of Applied Sciences, Breda, the Netherlands

**Keywords:** Conspiracy theories, health-related beliefs, longitudinal design, physical distancing, SARS-CoV2

## Abstract

**Background:**

Little is known about how conspiracy beliefs and health responses are interrelated over time during the course of the coronavirus disease 2019 (Covid-19) pandemic. This longitudinal study tested two contrasting, but not mutually exclusive, hypotheses through cross-lagged modeling. First, based on the consequential nature of conspiracy beliefs, we hypothesize that conspiracy beliefs predict an increase in detrimental health responses over time. Second, as people may rationalize their behavior through conspiracy beliefs, we hypothesize that detrimental health responses predict increased conspiracy beliefs over time.

**Methods:**

We measured conspiracy beliefs and several health-related responses (i.e. physical distancing, support for lockdown policy, and the perception of the coronavirus as dangerous) at three phases of the pandemic in the Netherlands (*N* = 4913): During the first lockdown (Wave 1: April 2020), after the first lockdown (Wave 2: June 2020), and during the second lockdown (Wave 3: December 2020).

**Results:**

For physical distancing and perceived danger, the overall cross-lagged effects supported both hypotheses, although the standardized effects were larger for the effects of conspiracy beliefs on these health responses than vice versa. The within-person change results only supported an effect of conspiracy beliefs on these health responses, depending on the phase of the pandemic. Furthermore, an overall cross-lagged effect of conspiracy beliefs on reduced support for lockdown policy emerged from Wave 2 to 3.

**Conclusions:**

The results provide stronger support for the hypothesis that conspiracy beliefs predict health responses over time than for the hypothesis that health responses predict conspiracy beliefs over time.

The coronavirus disease 2019 (Covid-19) pandemic has stimulated a surge of conspiracy theories, including beliefs that the virus was created by the pharmaceutical industry, that telecommunication companies have caused the pandemic with 5G radiation, or that China has created the virus to pursue geopolitical goals. Conspiracy theories gain traction in times of societal crisis such as war, societal unrest, economic downturn, floods, pandemics, or climate change (Van Prooijen & Douglas, 2017). Conspiracy theories are commonly defined as explanatory beliefs that a group of actors colludes in secret agreement to pursue goals widely seen as malevolent (Bale, 2007; for overviews, see Douglas et al., 2019; Van Prooijen, 2018, 2020; Van Prooijen & Van Vugt, 2018). Covid-19 conspiracy theories are not harmless. Belief in such theories is associated with detrimental health beliefs and behaviors such as decreased physical distancing, underestimating the dangers of the virus, unsubstantiated beliefs to already have experienced infection, lower vaccination intentions, and opposition against lockdown policies (Freeman et al., 2022; Hornsey et al., 2021; Imhoff & Lamberty, 2020; Marinthe, Brown, Delouvée, & Jolley, 2020; Pummerer et al., 2022; Van Prooijen, Etienne, Kutiyski, & Krouwel, 2021, 2022).

While some crisis events that stimulate conspiracy theories take place within a limited time window (e.g. a terrorist strike), the Covid-19 pandemic is a dynamic, ongoing event characterized by fluctuations in infection and hospitalization rates, virus mutations, changes in travel restrictions, lockdown policies, and so on. This extended temporal nature of the pandemic provides a unique opportunity to investigate the relationships between Covid-19 conspiracy beliefs and various health-related beliefs and behaviors over time (Bierwiaczonek, Gundersen, & Kunst, 2022; Bierwiaczonek, Kunst, & Pich, 2020; Hornsey et al., 2021; Pummerer et al., 2022). The present study sought to address the dynamic relationship between conspiracy beliefs and health responses on a large Dutch panel (*N* = 4913) at three time points during the pandemic: during the first infection peak and lockdown (April 2020), in between lockdowns (June 2020), and during the second lockdown (December 2020). At each time point, we measured participants' beliefs in conspiracy theories and various health-related responses during the pandemic, notably physical distancing, support for lockdown policies, and the perception of the virus as dangerous.

A previous project based on the same larger dataset descriptively revealed that conspiracy beliefs in April 2020 predicted a range of (mostly binary) outcome measures in December 2020 (e.g. Did people get tested for corona, and if so, was the test positive? Did people lose their jobs? Van Prooijen et al., 2021). The present contribution sought to meaningfully extend this project by examining the temporal directionality of the relationship between conspiracy beliefs and health responses through testing their cross-lagged effects over the three waves. This approach is uniquely suited to test two contrasting, but not mutually exclusive hypotheses of the temporal relationship between conspiracy beliefs and health responses: (1) conspiracy beliefs predict a progressive increase in detrimental health beliefs and behaviors over time (which we call the *Consequential Conspiracy Theories Hypothesis*), and (2) detrimental health beliefs and behaviors predict a progressive increase in conspiracy beliefs over time (which we call the *Rationalizing Conspiracy Theories Hypothesis*).

## The consequential conspiracy theories hypothesis

One basic property of conspiracy theories is that they are consequential: What people believe drives their subsequent perceptions and behaviors (Van Prooijen & Douglas, 2018; Van Prooijen & Van Vugt, 2018). Experimental research has underscored that conspiracy theories influence people's perceptions and behaviors in the context of health, relationships, and society. For instance, exposing people to anti-vaccine conspiracy theories lowers their willingness to get a fictitious child vaccinated (Jolley & Douglas, 2014a), and exposing people to climate change conspiracy theories decreases their willingness to lower their carbon footprints (Jolley & Douglas, 2014b). Conspiracy theories increase people's willingness to commit minor forms of crime (e.g. filing false insurance claims; Jolley, Douglas, Leite, and Schrader, 2019), deteriorate social relationships (Van Prooijen, Spadaro, & Wang, 2022), and, in the context of the Covid-19 pandemic, reduces people's support of regulations to contain the virus (Pummerer et al., 2022).

It should be emphasized that the current study cannot prove causality, as longitudinal designs cannot control for all possible confounding variables (e.g. Ployhart and Ward, 2011). Yet, based on the notion that conspiracy theories are consequential, we can theorize how the link between conspiracy beliefs and health responses might develop over time. Specifically, conspiracy theories erode the trust that people have in health authorities (e.g. Freeman et al., 2022; Karic and Mededovic, 2021; Pummerer et al., 2022; Šrol, Mikušková, & Čavojová, 2021; Van Prooijen, Etienne, Kutiyski, & Krouwel, 2022), and hence, belief in conspiracy theories might progressively lower people's willingness to implement health recommendations of those authorities. Consistent with this reasoning, a preliminary longitudinal study over a short time span (mid-March to mid-April 2020) revealed that conspiracy beliefs predicted decreased physical distancing in subsequent waves (Bierwiaczonek et al., 2020). Hence, the consequential hypothesis stipulates that conspiracy beliefs are associated with a progressive decrease over time in physical distancing, support for lockdown policy, and a perception of the virus as dangerous.

## The rationalizing conspiracy theories hypothesis

An alternative possibility, however, is that the association between health responses and conspiracy beliefs emerges through a reverse temporal order: Detrimental health beliefs and behaviors may predict an increase in conspiracy beliefs over time. Such a temporal order follows from the idea that people use conspiracy theories to justify and rationalize their existing beliefs and behaviors (Mercier, 2020). This line of reasoning is consistent with psychological theories of cognitive dissonance, stipulating that people often try to justify their beliefs and behaviors – to themselves and others – when they are incompatible with other beliefs or prevailing social norms (Festinger, 1957). Throughout the pandemic there were strong norms to follow the guidelines of health authorities, rendering it plausible that people experienced a high need to justify an unwillingness to adhere to these guidelines.

Conspiracy theories help people understand their social environment (Douglas et al., 2019; Van Prooijen, 2020). Recent findings have suggested that this sense-making function extends towards self-perception: Conspiracy beliefs predict an increased likelihood of misinterpreting one's own physical discomfort as evidence of a Covid-19 infection, reinforcing a perception of the coronavirus as not dangerous (Van Prooijen et al., 2022). Also in politics, people believe conspiracy theories as the result of a motivated reasoning process to support their ideologies (Enders & Smallpage, 2019; Miller, Saunders, & Farhart, 2016), and accordingly, people may use conspiracy theories as a tool to justify their beliefs and behaviors across various life domains (Van Prooijen, 2022). In sum, the rationalizing hypothesis predicts that low levels of physical distancing, low support for lockdown policies, and low danger perceptions predict a progressive increase in conspiracy beliefs over time.

## Method

### Participants and design

The study had a longitudinal design on a large research panel in the Netherlands. Wave 1 took place during the first lockdown (April 2020, *N* = 9033); Wave 2 took place after the first lockdown, when many restrictions were lifted although physical distancing was still mandatory (June 2020, *N* = 6775); Wave 3 took place during the second lockdown (December 2020, *N* = 5745). A total of 4913 respondents participated in all three waves; this sample forms the basis of the present analyses (3516 men, 1397 women; *M*_age_ = 59.68, s.d. = 14.45; analysis of attrition in the Online Supplemental Materials). Sample size in the analyses deviates from the total sample size due to missing values. Online Supplemental Materials (OSM) and an anonymized copy of the data and analysis code to reproduce the results are available on OSF (https://osf.io/bn6xe/). The study was not preregistered.

### Procedure

Data were collected by Kieskompas (‘Election compass’), a Dutch political research organization that coordinates large research panels. Kieskompas complies with EU privacy (GDPR) regulations, is closely monitored by the Dutch privacy authority, and adheres to the ethical norms of Vrije Universiteit Amsterdam. The panels were acquired through Voting Advice Applications prior to Dutch elections, and were complemented with targeted survey studies. For each wave, participants were invited through email. The study was part of a larger research project on the psychological, moral, and political processes underlying human behavior during the Covid-19 pandemic (Krouwel, Etienne, & Kutiyski, 2020; see also Van Prooijen et al., 2021, 2022).

### Measures

The measures that form the basis of the present contribution were assessed in all three waves (overview of items in the OSM). To measure *conspiracy beliefs*, participants indicated how credible they found nine statements referring to pandemic-related conspiracy theories that were common in the Netherlands in 2020 (1 = *not very credible*; 5 = *very credible*), including ‘The virus has been released by the US government to destabilize China’ and ‘The virus was developed by pharmaceutical companies’ (Van Prooijen et al., 2022; Wave 1: *α* = 0.84; Wave 2: *α* = 0.85; Wave 3: *α* = 0.86; Descriptive statistics of the conspiracy theory items in the OSM, Online Supplementary Table S1).^*^^1^

To measure *physical distancing*, we assessed four items on an 11-point slider (0 = *strongly disagree*, 10 = *strongly agree*). For example, participants indicated whether at this point in the pandemic they stayed at home as much as possible (Van Bavel et al., 2022; Wave 1: *α* = 0.69; Wave 2: *α* = 0.72; Wave 3: *α* = 0.72).^2^

We assessed participants' *support for lockdown policy* with five items (0 = *strongly disagree*, 10 = *strongly agree*). For example, participants indicated whether at this point in the pandemic they supported closing down bars and restaurants (Van Bavel et al., 2022; Wave 1: *α* = 0.79; Wave 2: *α* = 0.82; Wave 3: *α* = 0.79).

Finally, we measured how dangerous participants perceived the virus to be, with three items (1 = *certainly not*, 5 = *certainly*), e.g. ‘It is dangerous to get infected with the coronavirus’ (Van Prooijen et al., 2022; Wave 1: *α* = 0.63; Wave 2: *α* = 0.68; Wave 3: *α* = 0.74).

## Results

### Analysis

The descriptive statistics and intercorrelations of the measured variables are displayed in Table 1. The data were analyzed through structural equation modeling, using the *lavaan*-package in R (Rosseel, 2012). To establish model fit we relied on the three most common indicators, notably the CFI (reflecting acceptable fit if >0.90), the RMSEA (<0.08) and the SRMR (<0.08).

**Table 1.** Descriptive statistics and intercorrelations of the measured variables

	*M*	s.d.	Med.	Min.	Max.	1	2	3	4	5	6	7	8	9	10	11
1. Conspiracy beliefs W1	1.72	0.54	1.44	1.00	4.89	–										
2. Conspiracy beliefs W2	1.77	0.69	1.33	1.00	4.44	0.69	–									
3. Conspiracy beliefs W3	1.73	0.65	1.44	1.00	4.89	0.70	0.77	–								
4. Physical distancing W1	7.99	2.08	8.50	0.00	10.00	−0.14	−0.28	−0.29	–							
5. Physical distancing W2	7.22	2.22	7.75	0.00	10.00	−0.20	−0.43	−0.42	0.55	–						
6. Physical distancing W3	7.79	2.12	8.25	0.00	10.00	−0.17	−0.42	−0.37	0.54	0.66	–					
7. Support for policy W1	6.85	2.30	7.00	0.00	10.00	−0.13	−0.09	−0.11	0.60	0.43	0.36	–				
8. Support for policy W2	4.52	2.32	4.40	0.00	10.00	−0.06	−0.12	−0.19	0.37	0.54	0.39	0.55	–			
9. Support for policy W3	6.02	2.35	6.40	0.00	10.00	−0.12	−0.23	−0.29	0.34	0.45	0.56	0.45	0.54	–		
10. Perceived danger W1	3.60	0.86	3.67	1.00	5.00	−0.22	−0.25	−0.25	0.39	0.51	0.34	0.48	0.40	0.30	–	
11. Perceived danger W2	3.55	0.97	3.67	1.00	5.00	−0.24	−0.39	−0.42	0.32	0.59	0.53	0.37	0.50	0.49	0.76	–
12. Perceived danger W3	3.72	0.98	4.00	1.00	5.00	−0.28	−0.39	−0.40	0.42	0.54	0.57	0.42	0.45	0.58	0.62	0.73

*Note*: Conspiracy beliefs and perceived danger are measured on five-point scales (1–5), physical distancing and support for lockdown policy are measured on 11-point scales (0–10). All correlations were significant (*p* < 0.001).

The most common statistical method to establish reciprocal relationships between two variables over time – in our case, conspiracy beliefs and health responses to the Covid-19 pandemic – is the cross-lagged panel model (CLPM). This model simultaneously specifies the cross-sectional relationships between the two variables, the autoregressive (stability) relationships of the same variable over time, and the cross-lagged relationships between these variables. The CLPM is hence designed to establish the temporal directionality of the relationship between two variables.

The traditional CLPM fails to separate within-person *v.* between-person variance in these relationships, however. A common alternative, therefore, is the random intercepts cross-lagged panel model (RI-CLPM; Hamaker, Kuiper, and Grasman, 2015; Mulder and Hamaker, 2021). The RI-CLPM controls for stable differences between participants by including a random intercept in the model. As such, the RI-CLPM provides information about the unique influence of a predictor variable (i.e. after partialling out the variance of stable individual differences) on the extent to which individual participants have changed over time on the outcome variable. It can therefore yield substantially different results than the CLPM. These features of the RI-CLPM also imply a limitation, however, in that it only focuses on temporal fluctuations around the mean responses within individual participants. Quite often the factors that cause between-person differences over time are of central interest in longitudinal research questions. Moreover, the RI-CLPM does not control for third-variable confounders that change over time (Lüdtke & Robitzsch, 2021, july 29; Mund, Johnson, & Nestler, 2021; Orth, Clark, Donnellan, & Robins, 2020; but see Lucas, 2022, february 14).

**Fig. 1.**
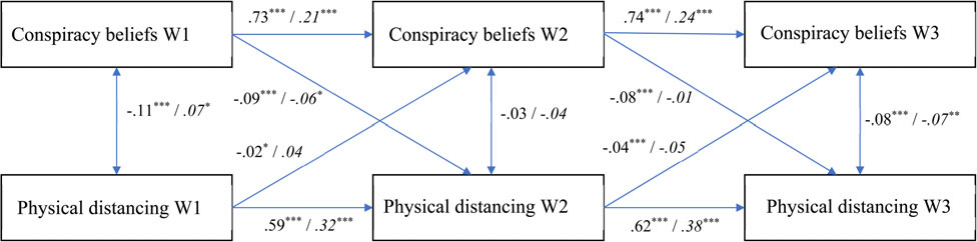
The relationship between conspiracy beliefs and physical distancing over time (fully standardized solution). Values at the left represent the CLPM, values at the right (and in italics) represent the RI-CLPM. * *p* < 0.05; ** *p* < 0.01; *** *p* < 0.001.

**Fig. 2.**
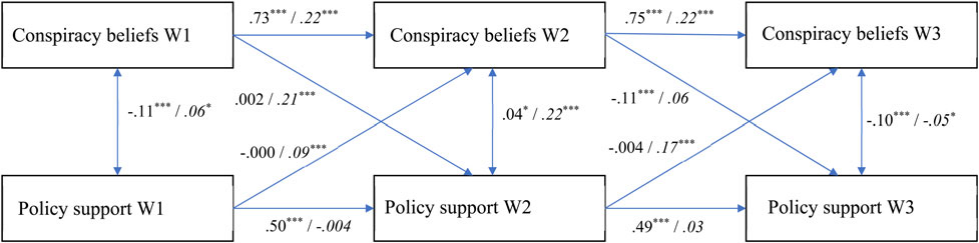
The relationship between conspiracy beliefs and support for lockdown policy over time (fully standardized solution). Values at the left represent the CLPM, values at the right (and in italics) represent the RI-CLPM. * *p* < 0.05; ** *p* < 0.01; *** *p* < 0.001.

**Fig. 3.**
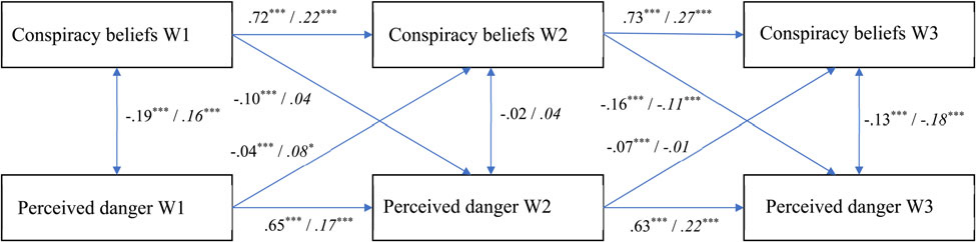
The relationship between conspiracy beliefs and perceived danger over time (fully standardized solution). Values at the left represent the CLPM, values at the right (and in italics) represent the RI-CLPM. * *p* < 0.05; ** *p* < 0.01; *** *p* < 0.001.

In the current study, we therefore used the CLPM and RI-CLPM in a complementary fashion. For each health response (physical distancing, support for lockdown policy, and perceived danger of the coronavirus) we first analyzed the CLPM to assess its overall cross-lagged effects with conspiracy beliefs. Subsequently, we also assessed the RI-CLPM to establish the extent to which these effects are due to within-person changes as the pandemic progressed. Figures 1–3 display the fully standardized solutions of these models; unstandardized estimates (*B*s), standard errors, and confidence intervals are in the OSM (Online Supplementary Table S2).

### Physical distancing

The CLPM had a good fit to the data according to two out of three indicators [CFI = 0.934; RMSEA = 0.211, CI_90%_(0.199–0.224); SRMR = 0.047; χ^2^(4, *N* = 4118) = 739.47, *p* < 0.001]. Only the RMSEA suggested a poor fit. Monte Carlo simulations revealed, however, that the RMSEA often falsely indicates a poor fit when models have small degrees of freedom (Kenny, Kaniskan, & McCoach, 2015). Given the CFI and SRMR, we consider the fit acceptable. As indicated in Fig. 1, conspiracy beliefs and physical distancing were significantly negatively associated when there was a lockdown (Waves 1 and 3) but not when there was no lockdown (Wave 2). All the cross-lagged effects were significant. Stronger conspiracy beliefs at a particular wave predicted a decrease in physical distancing at the next wave, and physical distancing at a particular wave predicted a decrease in conspiracy beliefs at the next wave. The coefficients were significantly larger for the effects of conspiracy beliefs on physical distancing over time, rather than vice versa, Wald χ^2^(2) = 81.48, *p* < 0.001.

More generally, the standardized coefficients for the paths from physical distancing to conspiracy beliefs were rather low. A Monte Carlo power analysis for parameter values (Wang & Rhemtulla, 2021; 1000 simulations) – given the current sample size and specifying all other coefficients in the model as population values – revealed that while the power to detect the standardized coefficient from physical distancing T2 to conspiracy beliefs T3 (−0.04) was high (power = 0.99), the power to detect the coefficient from T1 to T2 (−0.02) was low (power = 0.42). Our sample hence allowed for a high probability to detect small effect sizes.

We then assessed the RI-CLPM to establish within-person changes over time. The model fitted the data well [CFI = 1.00; RMSEA = 0.063, CI_90%_(0.040–0.091); SRMR = 0.012; χ^2^(1, *N* = 4118) = 17.56, *p* < 0.001]. The random intercepts (reflecting temporally stable between-subjects variance) of conspiracy beliefs and physical distancing were negatively associated [Estimate = −0.094, s.e. = 0.010; *z* = −8.987, *p* < 0.001; CI_95%_(−0.114 to −0.073)]. The autoregressive effects for both conspiracy beliefs and physical distancing were smaller than in the CLPM; this is to be expected, as the temporally stable, trait-like variance is separated from these effects in the RI-CLPM. Hence, these autoregressive effects represent within-person carry-over effects (Hamaker et al., 2015; Mulder & Hamaker, 2021). The covariances between conspiracy beliefs and physical distancing at a particular wave in the RI-CLPM represent the relationship between the within-person residuals of these variables.

Of central interest in the RI-CLPM are the cross-lagged effects. The standardized coefficients indicate that stronger conspiracy beliefs significantly predict a decrease in physical distancing over time, but only early in the pandemic (i.e. from Wave 1 to Wave 2; see Fig. 1). In other words, conspiracy beliefs at Wave 1 uniquely predicted within-person decreases in physical distancing from Wave 1 to Wave 2; subsequent within-person changes in physical distancing (from Wave 2 to Wave 3) did not depend on conspiracy beliefs. The cross-lagged effects from physical distancing to conspiracy beliefs were not significant.

We then also tested a nested model constraining the lagged effects in the RI-CLPM to be equal over time (Mulder & Hamaker, 2021).^3^ This model fitted the data significantly worse, Δχ^2^(4) = 17.56, *p* = 0.002, providing further evidence for within-person change over time.

### Support for lockdown policy

The CLPM had an acceptable fit according to two out of three indicators, again except for the RMSEA [CFI = 0.90; RMSEA = 0.241, CI_90%_(0.228–0.254); SRMR = 0.064; χ^2^(4, *N* = 4118) = 959.48, *p* < 0.001]. The cross-sectional relationships between conspiracy beliefs and support for lockdown policy were significant and negative during the two lockdowns (Waves 1 and 3). At Wave 2, this link was (weakly) positive, which might be due to the fact that during the second wave most restrictions had been lifted, and not many people supported lockdown policy. Indeed, the means for this measure were substantially lower during Wave 2 than Waves 1 or 3 (see Table 1). As to the cross-lagged effects, conspiracy beliefs at Wave 2 predicted a decreased support for lockdown policy at Wave 3. None of the other cross-lagged effects were significant.

The RI-CLPM, then, fitted the data well [CFI = 1.00; RMSEA = 0.061, CI_90%_(0.037–0.089); SRMR = 0.012; χ^2^(1, *N* = 4118) = 16.39, *p* < 0.001]. The random intercepts of conspiracy beliefs and support for lockdown policy were negatively associated [Estimate = −0.119, s.e. = 0.012; *z* = −9.711, *p* < 0.001; CI_95%_(−0.143 to −0.095)], reflecting the basic finding that conspiracy beliefs predict decreased support for lockdown policy. The results revealed various significant cross-lagged effects (see Fig. 2): From conspiracy beliefs at Wave 1 to support for lockdown policy at Wave 2, and from support for lockdown policy at Waves 1 and 2 to conspiracy beliefs at Waves 2 and 3. Paradoxically, however, these cross-lagged effects were *positive*, suggesting that conspiracy beliefs were associated with increased support for lockdown policy over time, and vice versa. A nested model constraining the lagged effects to be equal over time had a worse fit to the data, Δχ^2^(4) = 13.759, *p* = 0.008, supporting these within-person changes over time.

These positive effects may be understood by taking into account that (a) they reflect within-person changes only, and (b) there were few restrictions in June 2020 with corresponding low overall support for a lockdown during Wave 2 (see Table 1). For example, if people low in conspiracy beliefs mostly support governmental regulations, they may show a relatively large decrease in support for lockdown policy from Wave 1 (when many restrictions were in place) to Wave 2 (when few restrictions were in place). This within-person change may be less pronounced among people high in conspiracy beliefs, who may be less likely to adjust their views depending on changing circumstances. Altogether, the somewhat counterintuitive within-person change results for this variable are most likely due to the fact that a lockdown was neither in place nor supported by large portions of the Dutch public during Wave 2.

### Perceived danger

For the perceived danger of the coronavirus (Fig. 3), the CLPM had a good fit, again according to the CFI and SRMR but not according to the RMSEA [CFI = 0.93; RMSEA = 0.224, CI_90%_(0.211–0.237); SRMR = 0.048; χ^2^(4, *N* = 4118) = 829.37, *p* < 0.001]. The results largely mirrored those for social distancing: The cross-sectional links between conspiracy beliefs and perceived danger were significant (and negative) during lockdowns (Waves 1 and 3) but not in-between lockdowns (Wave 2). All cross-lagged effects between conspiracy beliefs and perceived danger were significant, although again the coefficients were larger for the effects of conspiracy beliefs on perceived danger rather than vice versa, Wald χ^2^(2) = 173.62, *p* < 0.001.

The RI-CLPM had a good fit according to the CFI and the SRMR, although the RMSEA was marginal [CFI = 1.00; RMSEA = 0.094, CI_90%_(0.070–0.121); SRMR = 0.017; χ^2^(1, *N* = 4118) = 37.45, *p* < 0.001]. The random intercepts of conspiracy beliefs and perceived danger showed a negative relationship, reflecting that the more strongly people believe conspiracy theories, the less dangerous they perceive the virus to be [Estimate = −0.086, s.e. = 0.006; *z* = −15.244, *p* < 0.001; CI_95%_(−0.097 to −0.075)]. Early in the pandemic, perceiving the virus as dangerous was associated with stronger conspiracy beliefs over time. Perceived danger at Wave 1 uniquely predicted a within-person increase in conspiracy beliefs from Wave 1 to Wave 2. Later in the pandemic, however, conspiracy beliefs predicted a reduced perception of the virus as being dangerous. Conspiracy beliefs at Wave 2 uniquely predicted a within-person decrease in perceived danger from Wave 2 to Wave 3. These within-person change effects are further supported by the finding that a nested model constraining the lagged effects to equality fitted the data worse, Δχ^2^(4) = 27.425, *p* < 0.001.

## Discussion

The present study investigated the dynamic temporal relationship between conspiracy beliefs and health responses by soliciting three waves during the first year of the Covid-19 pandemic in the Netherlands. This longitudinal design enabled a test of two possible explanations of the relationship between conspiracy beliefs and detrimental health beliefs and behaviors, The Consequential Conspiracy Theories Hypothesis and the Rationalizing Conspiracy Theories Hypothesis. We interpret the results in the context of these two theoretical ideas.

For physical distancing, the results provide stronger support for the consequential than for the rationalizing hypothesis. Although the overall cross-lagged effects provided some support for both hypotheses, the standardized effects of conspiracy theories predicting physical distancing over time were larger than the reverse pattern. In terms of within-participant change, conspiracy theories predicted a decrease in physical distancing, but only early in the pandemic – presumably, later in the pandemic physical distancing behavior may have stabilized within individuals, or was determined by other factors (e.g. face mask mandates in public places). These results are consistent with other research, indicating that conspiracy beliefs predict a progressive decrease in physical distancing (Bierwiaczonek et al., 2020), and that Covid-19 conspiracy beliefs decrease people's support for restrictive measures (Pummerer et al., 2022).

On support for lockdown policies, results offered some evidence for the consequential and not the rationalizing hypothesis, but with some qualifications. Notably, conspiracy beliefs predicted an overall decrease in support for lockdown policy over time, but only in the period from June 2020 (when there was no lockdown) to December 2020 (the second lockdown). Presumably, this effect did not emerge from Wave 1 to Wave 2 given the low overall support for lockdown policies in June 2020, when the Dutch government had reopened many parts of society (e.g. bars/restaurants, culture, sports, and so on). Most likely due to these relaxations of restrictions that changed societal reality, patterns of support for lockdown policies also shifted, making the within-person change results for this variable somewhat difficult to interpret.

The overall cross-lagged effects for perceiving the coronavirus as dangerous were similar to those for physical distancing. The results supported the consequential hypothesis more strongly than the rationalizing hypothesis. On the overall cross-lagged effects, the standardized effects of conspiracy beliefs on perceived danger over time were larger than those in the reverse pattern. The within-person change results revealed a dynamic interplay between these variables over time. Early in the pandemic (from Wave 1 to Wave 2), perceived danger predicted a within-person *increase* in conspiracy beliefs. As opposed to a rationalization process (Mercier, 2020; Van Prooijen, 2022), this finding is consistent with the notion that feelings of existential threat increase conspiracy beliefs (Douglas et al., 2019; Van Prooijen, 2020; Van Prooijen & Douglas, 2017). Later in the pandemic (from Wave 2 to 3), however, conspiracy beliefs predicted a within-person decrease in perceiving the virus as dangerous. Perhaps people increasingly became habituated to the health crisis, and hence experienced decreased existential threat later in the pandemic. Moreover, the link with perceived danger differs across conspiracy theories (e.g. Covid-19 as bioweapon *v.* hoax; Imhoff and Lamberty, 2020), and it is possible that conspiracy theories that downplayed the dangers of the virus became increasingly prominent as the pandemic progressed.

Taken together, the results are mostly consistent with the consequential hypothesis: Conspiracy beliefs predicted more detrimental health beliefs and behaviors over time, although within-person changes differed across outcome variables and depended on the specific phase of the pandemic. It would be premature to discard the idea that conspiracy beliefs can serve to rationalize one's health beliefs and behaviors, however (see also Bierwiaczonek et al., 2022). The overall cross-lagged effects offered (weak) support for this perspective on physical distancing and perceived danger. Moreover, the present research did not examine an exhaustive list of health beliefs and behaviors. Future research might determine if people rationalize other important health behaviors (e.g. vaccination) through conspiracy theories.

While the present research has a number of strengths – such as the large sample size and the longitudinal design – it also has a number of limitations. First, the results were shaped by idiosyncrasies of how the pandemic – and relevant governmental policies – has unfolded in the Netherlands. The pandemic has elicited different policies across countries, however, ranging from strict lockdowns (e.g. New Zealand) to keeping society open as much as possible (e.g. Sweden). While the Netherlands has endorsed policy largely consistent with many other EU countries (and arguably therefore is a good case study), it is unclear to what extent the present findings would generalize to countries with different policies. Second, while the results were mostly consistent with the idea that conspiracy theories are consequential, longitudinal designs do not offer conclusive evidence about causality (Ployhart & Ward, 2011). The current findings hence need to be complemented by experimental designs showing that conspiracy beliefs causally shape health beliefs and behaviors (Jolley & Douglas, 2014a; Pummerer et al., 2022). Finally, not all psychometric qualities of our data were optimal. For instance, men were overrepresented. Although it is unclear what this implies for the results, previous research did not find reliable gender effects on Covid-19 conspiracy beliefs (e.g. Freeman et al., 2022). Moreover, belief in conspiracy theories was low in the sample. Future research may therefore focus on more general conspiracy theories (e.g. beliefs that the government is not telling the truth) that are likely to be more widespread in society (Hornsey et al., 2021).

These limitations notwithstanding, there is a paucity of studies examining the longitudinal relationships between conspiracy beliefs and health beliefs and behaviors. Particularly in the context of the Covid-19 pandemic – where health responses such as physical distancing directly influence the likelihood of infection with a dangerous virus (Fazio et al., 2021) – it is important to understand how these health responses develop over time. The present findings underscore the role of conspiracy beliefs for detrimental health responses in the context of Covid-19, not only in the short run (e.g. Freeman et al., 2022; Imhoff and Lamberty, 2020; Marinthe et al., 2020) but also in the long run (see also Van Prooijen et al., 2021). Apparently, conspiracy theories predict deteriorated health responses to Covid-19 throughout the course of the pandemic.

## Footnotes

* The notes appear after the main text.

## Notes

1 The questionnaire also contained a different, 4-item measure of conspiracy beliefs measured on an 11-point scale (Van Prooijen et al., 2021). As reported conspiracy beliefs were very low on this scale, we report the results for this measure in the OSM.

2 The scale also contained a fifth (recoded) item, referring to having visited friends, family, and colleagues outside of one's household. We have dropped this item to improve the scale's reliability particularly in Wave 1 (*α* = 0.61 with the item included) and Wave 3 (*α* = 0.69 with the item included).

3 We thank an anonymous reviewer for recommending this additional analysis.

## Supplementary material

For supplementary material accompanying this paper visit https://doi.org/10.1017/S0033291722002938. click here to view supplementary material
