# Supplementary material for: A longitudinal analysis of conspiracy beliefs and Covid-19 health responses
Source: Psychol Med. 2022 Sep 26;53(12):5709–16. doi: 10.1017/S0033291722002938 (PMC10482717; doi:10.1017/S0033291722002938)
Supplement: Supplementary file 1 [file S0033291722002938sup001.docx]

Online Supplementary Materials

For

**A Longitudinal Analysis of Conspiracy Beliefs and Covid-19 Health Responses**

Jan-Willem van Prooijen^1,2,3^, David Amodio^4,5^, Arnout Boot^6^, Anita Eerland^7^, Tom Etienne^8,9^, André P. M. Krouwel^1^, Michal Onderco^6,10^, Peter Verkoeijen^6,11^, and Rolf A. Zwaan^6^

^1^ Vrije Universiteit Amsterdam, the Netherlands

^2^ The Netherlands Institute for the Study of Crime and Law Enforcement (NSCR), the Netherlands

^3^ Maastricht University, the Netherlands

^4^ New York University, USA

^5^ University of Amsterdam, the Netherlands

^6^ Erasmus University Rotterdam, the Netherlands

^7^ Radboud University Nijmegen, the Netherlands

^8^ Kieskompas, the Netherlands

^9^ University of Pennsylvania, PA, USA

^10^ Peace Research Center Prague, Czech Republic

^11^ Avans University of Applied Sciences

**Measures**

**Covid-19 Conspiracy beliefs**

How credible do you find the below statements about the corona virus? (1 = not very credible, 5 = very credible)

1. The virus has been released by the US government to destabilize China
2. The virus was developed to control population growth
3. The virus is a way to cover up the effects of 5G towers
4. The virus was developed by pharmaceutical companies
5. Together with the future vaccine, a chip will be injected to permanently track people
6. The Chinese government lies about the number of corona deaths in China
7. The corona virus was spread deliberately among the Chinese population
8. The corona virus was developed by the Chinese government to damage the Western world and its economies, in order to become the strongest economy in the world
9. The corona virus was developed by climate activists to counteract climate change

*As is common procedure in conspiracy theory research, in the manuscript these items were averaged to form a reliable scale for the analyses. In Table S1 below, however, more detailed information is provided about the descriptive statistics of these individual Covid-19 Conspiracy Beliefs items.*

**Physical distancing:**

*Responses on a slider, ranging from 0 = strongly disagree, 10 = strongly agree*)

During the days of the corona pandemic……

- I stay at home as much as possible
- I have visited friends, family or colleagues outside my home (recoded)
- I limit the number of visits to the supermarket to an absolute minimum
- I keep physical distance from other people outside my home
- I avoid shaking hands with people outside my home

**Support for lockdown policy:**

*Responses on a slider, ranging from 0 = strongly disagree, 10 = strongly agree*)

During the days of the corona pandemic, I am in favor of……

- Closing down all schools and universities
- Closing down all bars and restaurants
- Closing down all parks
- Prohibiting all public gatherings where a lot of people get together at the same place (sports and culture)
- Prohibiting all unnecessary traveling

**Perceived danger:**

*Responses on a scale (1 = certainly not, 5 = certainly)*

- It is dangerous to get infected with the corona virus
- Do you believe that many citizens will die as a consequence of Covid-19?
- Do you believe that the media exaggerate the dangers of Covid-19? (recoded)

**Analysis of attrition**

To test whether attrition was random or systematic, we statistically compared participants who completed all three waves with participants who did not complete all three waves on the measured variables at Wave 1. A MANOVA yielded a significant multivariate effect, *F*(4, 8472) = 12.128, *p* < .001, but with a very small effect size, η^2^ = .006. Participants who completed all waves had slightly lower conspiracy beliefs (*M* = 1.57, *SD* = 0.47) than participants who had not completed all waves (*M* = 1.64, *SD* = 0.53), *F*(1, 8475) = 40.26, *p* < .001; η^2^ = .005. We also observed small differences for physical distancing and perceived danger, albeit with negligible effect sizes; for physical distancing: *F*(1, 8475) = 11.47, *p* < .001; η^2^ = .001; for perceived danger, *F*(1, 8475) = 6.19, *p* = .013; η^2^ = .001. For support for lockdown policy, the effect of attrition was not significant, *F*(1, 8475) = 1.90, *p* = .169; η^2^ = .000. Altogether, while we found some differences on the measured variables between participants who did versus did not complete all the waves, these differences were extremely small. Hence, we conclude that it is unlikely that attrition was systematic or otherwise problematic in this study.

**Table S1**

Means, Standard Deviations, Medians, Minimum and Maximum Values for the Individual Covid-19 Conspiracy Theory Items.

Item Wave 1 Wave 2 Wave 3

*M SD Med Min. Max. M SD Med Min. Max. M SD Med Min. Max.*

1. 1.21 0.57 1 1 5 1.22 0.59 1 1 5 1.20 0.52 1 1 5

2. 1.24 0.64 1 1 5 1.24 0.63 1 1 5 1.23 0.61 1 1 5

3. 1.12 0.48 1 1 5 1.11 0.42 1 1 5 1.13 0.45 1 1 5

4. 1.36 0.77 1 1 5 1.35 0.77 1 1 5 1.31 0.70 1 1 5

5. 1.33 0.71 1 1 5 1.29 0.70 1 1 5 1.16 0.54 1 1 5

6. 3.70 0.94 4 1 5 3.62 0.98 4 1 5 3.66 0.89 4 1 5

7. 1.50 0.78 1 1 5 1.47 0.77 1 1 5 1.55 0.78 1 1 5

8. 1.47 0.77 1 1 5 1.41 0.76 1 1 5 1.50 0.78 1 1 5

9. 1.19 0.49 1 1 5 1.17 0.47 1 1 5 1.21 0.53 1 1 5

*Note.* All items were measured on 5-point scales. Item wordings under “Measures” above.

**Table S2**

*B*s, *SE*s, and CIs of the cross-lagged paths in the CLPM and RI-CLPM models

*Path*  CLPM RI-CLPM

*B SE* CI_95%_ *B SE* CI_95%_

W1 conspiracy 🡪 W2 physical distancing -0.323 .047 -0.415; -0.231 -0.330 .163 -0.650; -0.009

W1 physical distancing 🡪 W2 conspiracy -0.007 .003 -0.014; -0.000 0.011 .008 -0.004; 0.025

W2 conspiracy 🡪 W3 physical distancing -0.256 .040 -0.335; -0.177 -0.031 .100 -0.226; 0.164

W2 physical distancing 🡪 W3 conspiracy -0.012 .003 -0.017; -0.006 -0.009 .005 -0.020; 0.001

W1 conspiracy 🡪 W2 policy support 0.008 .061 -0.112; 0.128 1.179 .195 0.796; 1.562

W1 policy support 🡪 W2 conspiracy -0.000 .003 -0.005; 0.005 0.018 .006 0.006; 0.029

W2 conspiracy 🡪 W3 policy support -0.480 .060 -0.599; -0.362 0.313 .165 -0.009; 0.636

W2 policy support 🡪 W3 conspiracy -0.004 .002 -0.008; 0.001 0.029 .005 0.018; 0.039

W1 conspiracy 🡪 W2 perceived danger -0.175 .022 -0.218; -0.133 0.089 .084 -0.076; 0.254

W1 perceived danger 🡪 W2 conspiracy -0.026 .006 -0.038; -0.013 0.049 .022 0.006; 0.091

W2 conspiracy 🡪 W3 perceived danger -0.293 .021 -0.335; -0.250 -0.217 .055 -0.324; -0.110

W2 perceived danger 🡪 W3 conspiracy -0.038 .006 -0.049; -0.027 -0.004 .014 -0.031; 0.022

**Additional conspiracy belief measure**

The questionnaire also contained a different, 4-item measure of conspiracy belief (Van Prooijen et al., 2021), that was impossible to integrate with the measure reported in the main manuscript as it was assessed on a different response scale (on a slider ranging from 0 to 10). As this variable yielded relatively low conspiracy beliefs across the three waves, we took the other, 9-item measure as our main indicator of conspiracy beliefs in the main manuscript. For full transparency, however, here we report the measure and the results also for this alternative, 4-item measure. The measure had the following items: “The coronavirus (COVID-19) is a bioweapon engineered by scientists”, “The coronavirus (COVID-19) is a conspiracy to take away citizens’ rights for good and establish an authoritarian government”, “The coronavirus (COVID-19) is a hoax invented by interest groups for financial gains”, and “The coronavirus (COVID-19) was created as a cover-up for the impending global economic crash” (Wave 1: α = .90; *M =* 0.68, *SD* = 1.41. Wave 2: α = .92; *M* = 0.75, *SD* = 1.50. Wave 3: α = .91; *M* = 0.60, *SD* = 1.38). On the following pages, we display the results (CLPM and RI-CLPM) using this alternative measure (Figures S1 to S3).

Conspiracy beliefs W1

Conspiracy beliefs W2

Conspiracy beliefs W3

Physical distancing W1

Physical distancing W2

Physical distancing W3

.58^***^ / *.31^***^*

-.08^***^ / *-.05^†^*

.72^***^ / *.33^***^*

-.03^**^ / -*.02*

-.09^***^ / *-.04^†^*

.61^***^ / *.37^***^*

.73^***^ / *.36^***^*

-.05^***^ / *-.08^**^*

-.14^***^ / *.01*

-.07^***^ / *-.09^***^*

-.14^***^ / *-.17^**^*

**Figure S1.** The relationship between the alternative, 4-item conspiracy beliefs measure and physical distancing over time (fully standardized solution). Values at the left represent the CLPM, values at the right (and in italics) represent the RI-CLPM. ^†^ *p* < .10;

^*^ *p* < .05; ^**^ *p* < .01; ^***^ *p* < .001.

CLPM: CFI = .935; RMSEA = .211, CI_90%_[.198; .224]; SRMR = .048; χ^2^[4, *N* = 4118] = 736.48, *p* < .001.

RI-CLPM: CFI = 1.00; RMSEA = .081, CI_90%_[.057; .108]; SRMR = .014; χ^2^[1, *N* = 4118] = 28.14, *p* < .001

Conspiracy beliefs W1

Conspiracy beliefs W2

Conspiracy beliefs W3

Policy support W1

Policy support W2

Policy support W3

.50^***^ / *-.009*

.02 / *.23^***^*

.72^***^ / *.33^***^*

-.001 / *.06^**^*

-.12^***^ / *.005*

.49^***^ / *.04*

.74^***^ / *.35^***^*

-.04^***^ / *.12^***^*

-.15^***^ / *.02*

.01 / *.15*^***^

-.14^***^ / *-.13^***^*

**Figure S2.** The relationship between the alternative, 4-item conspiracy beliefs measure and support for lockdown policy over time (fully standardized solution). Values at the left represent the CLPM, values at the right (and in italics) represent the RI-CLPM. ^*^ *p* < .05; ^**^ *p* < .01; ^***^ *p* < .001.

CLPM: CFI = 0.91; RMSEA = .237, CI_90%_[.224; .249]; SRMR = .063; χ^2^[4, *N* = 4118] = 925.64, *p* < .001.

RI-CLPM: CFI = 1.00; RMSEA = .024, CI_90%_[.000; .054]; SRMR = .005; χ^2^[1, *N* = 4118] = 3.36, *p* = .067.

Conspiracy beliefs W1

Conspiracy beliefs W2

Conspiracy beliefs W3

Perceived danger W1

Perceived danger W2

Perceived danger W3

.65^***^ / *.18^***^*

-.10^***^ / *.01*

.71^***^ / *.34^***^*

-.06^***^ / -*.02*

-.16^***^ / *-.15^***^*

.62^***^ / *.21^***^*

.72^***^ / *.38^***^*

-.08^***^ / *-.06^*^*

-.22^***^ / *.10^**^*

-.06^***^ / *-.06^*^*

-.18^***^ / *-.25^***^*

**Figure S3.** The relationship between the alternative, 4-item conspiracy beliefs measure and perceived danger over time (fully standardized solution). Values at the left represent the CLPM, values at the right (and in italics) represent the RI-CLPM. ^*^ *p* < .05; ^**^ *p* < .01; ^***^ *p* < .001.

CLPM: CFI = 0.93; RMSEA = .224, CI_90%_[.211; .237]; SRMR = .048; χ^2^[4, *N* = 4118] = 830.77, *p* < .001.

RI-CLPM: CFI = 1.00; RMSEA = .093, CI_90%_[.069; .120]; SRMR = .016; χ^2^[1, *N* = 4118] = 36.75, *p* < .001
